# The role of age and comorbidities in postoperative outcome of mitral valve repair: A propensity-matched study

**DOI:** 10.1097/MD.0000000000003938

**Published:** 2016-06-24

**Authors:** Vincent Bonnet, Clément Boisselier, Vladimir Saplacan, Annette Belin, Jean-Louis Gérard, Jean-Luc Fellahi, Jean-Luc Hanouz, Marc-Olivier Fischer

**Affiliations:** a Pôle Réanimations Anesthésie SAMU/SMUR, CHU de Caen, Avenue de la Côte de Nacre; b Department of Cardiac Surgery; c Department of Cardiology, University Hospital of Caen, Caen; d Department of Anaesthesiology and Critical Care, Hôpital Cardiologique Louis Pradel, Avenue du Doyen Lepine; e Faculty of Medicine, University of Lyon 1 Claude Bernard, Lyon; f EA 4650, Université de Caen Basse-Normandie, Esplanade de la Paix, CS, Caen, France.

**Keywords:** anesthesia, cardiac surgery, elderly subjects, geriatrics, postoperative complications

## Abstract

The average age of patients undergoing mitral valve repair is increasing each year. This retrospective study aimed to compare postoperative complications of mitral valve repair (known to be especially high-risk) between 2 age groups: under and over the age of 80.

Patients who underwent mitral valve repair were divided into 2 groups: group 1 (<80 years old) and group 2 (≥80 years old). Baseline characteristics, pre- and postoperative hemodynamic data, surgical characteristics, and postoperative follow-up data until hospital discharge were collected.

A total of 308 patients were included: 264 in group 1 (age 63 ± 13 years) and 44 in group 2 (age 83 ± 2 years). Older patients had more comorbidities (atrial fibrillation, history of cardiac decompensation, systemic hypertension, pulmonary hypertension, and chronic kidney disease) and they presented more postoperative complications (50.0% vs 33.7%; *P* = 0.043), with a longer hospital stay (8.9 ± 6.9 vs 6.6 ± 4.6 days; *P* = 0.005). To assess the burden of age, a propensity score was awarded to postoperative complications. Active smoking, chronic pulmonary disease, chronic kidney disease, associated ischemic heart disease, obesity, and cardio pulmonary by-pass duration were described as independent risk factors. When matched on this propensity score, there was no difference in morbidity or mortality between group 1 and group 2.

Older patients suffered more postoperative complications, which were related to their comorbidities and not only to their age.

## 1 Introduction

In most developed countries, life expectancy at the age of 80 has significantly increased and is expected to continue to increase within the next few decades. Among the various diseases associated with ageing, valvular heart diseases have been shown to be present in more than 10% of patients aged 75 and over.^[1]^ In past decades, progress in technology, surgery, and perioperative medicine have allowed elderly patients to benefit from surgery including cardiac surgery.^[2]^ As a result, mitral regurgitation surgery often combined with coronary revascularization or aortic valve surgery is a growing perioperative challenge for anesthesiologists, cardiologists, and surgeons since it carries a high risk in the elderly. Thus, postoperative mortality in elderly patients has been reported to range from 2.5% to 9.0%.^[2,3]^ Long-term survival was 68% but was associated with a high recurrence of heart failure, major bleeding, and stroke.^[4]^ It emphasizes the need for a preoperative risk assessment.

Age is a well-known independent risk factor for increased morbidity and mortality following cardiac surgery and mitral valve surgery^[5,6]^ and was therefore included in prognostic scores. Nevertheless, these data were published in the last decade and included a small number of octogenarian patients,^[7]^ the scores poorly reflecting the outcome, especially for medium-risk patients.^[8]^ The respective burden of age and its associated chronic or degenerative pathologies in postoperative outcomes is not clear and might affect the quality of preoperative risk assessment.

Consequently, the goal of this study was to examine the role of age and its associated morbidities in the postoperative outcome of mitral valve repair.

We hypothesized that morbidity rather than age itself may be the most important risk factor associated with postoperative outcome.

## 2 Methods

### 2.1 Patients

After agreement from the local ethics committee (Nord Ouest III CPP, University Hospital of Caen, Avenue de la Côte de Nacre, 14000 Caen, France; Hospital ref.: A14-D60-VOL.23, Chairman: Dr Charlotte Gourio), the medical records of all adults having undergone mitral valvuloplasty with cardiopulmonary bypass between February 2007 and December 2013 in Caen University Hospital, France were analyzed retrospectively. Exclusion criteria were emergency surgery (<24 hours after admission) or critical preoperative condition, defined by the occurrence of at least one of the following events: unstable angina requiring nitrate derivatives until surgery, ventricular tachycardia or fibrillation, resuscitated cardiac arrest, preoperative mechanical ventilation, preoperative use of vasopressors and/or intra-aortic balloon pump, or preoperative acute renal failure. Two groups of patients were defined: group 1 including patients under 80 years old and group 2 for those of 80 years old and over, according to the MeSH definition of oldest-old.

### 2.2 Parameters recorded

All data were collected using a predefined form including demographic data and past medical history, cardiovascular risk factors (gender, smoking, systemic hypertension, diabetes, hypercholesterolemia, and obesity), preoperative treatments, etiology of the mitral insufficiency, preoperative hemodynamic status (atrial fibrillation, left ventricular ejection fraction, pulmonary artery systolic pressure measured by cardiac ultrasonography, or right heart catheterization), surgical characteristics and perioperative data (cardiopulmonary bypass and aortic clamping duration), combined surgery (other valvular or coronary bypass surgery), number of associated coronary bypasses, and postoperative data collected in the intensive care unit.

### 2.3 Definitions of comorbidities and postoperative complications

Baseline serum creatinine was the last result before surgery. Chronic kidney disease was defined as a glomerular filtration rate lower than 60 mL/min/m^2^.^[9]^ Pulmonary arterial hypertension was defined as a pulmonary artery systolic pressure >50 mm Hg. Associated ischemic heart disease was defined as stenosis of more than 70% of a coronary artery.

Postoperative acute renal failure was defined, according to the risk, injury, failure, loss of kidney function, end-stage kidney disease classification, as a 25% decrease in glomerular filtration rate.^[10]^ Blood transfusion was defined as prescription of packed red blood cells within the first 48 hours after surgery.

### 2.4 Endpoints

**Figure 1:**
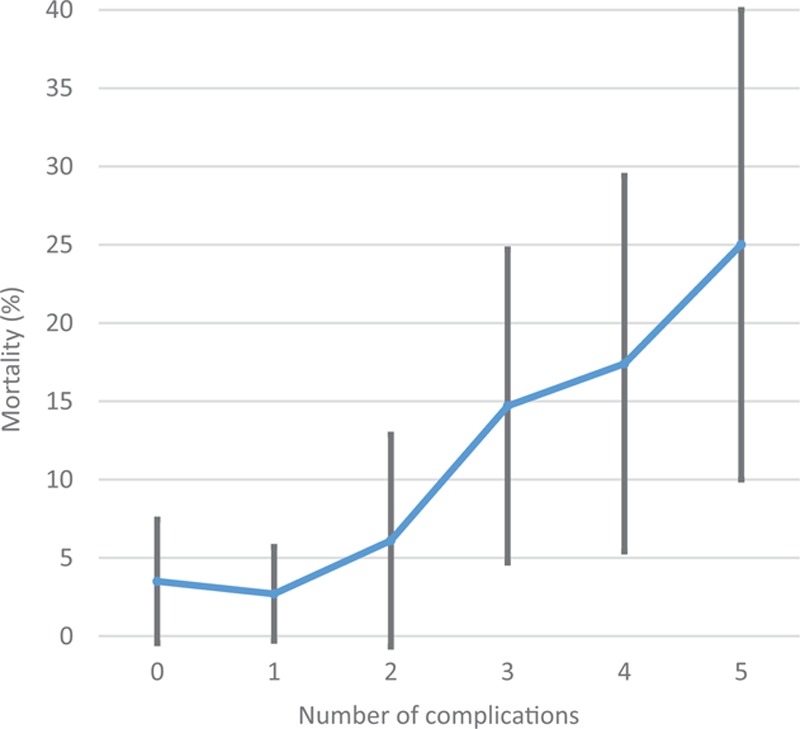
Mortality according to the number of postoperative complications.

Postoperative complications are associated with higher in-hospital^[11]^ and long-term mortality^[12]^ as well as longer hospitalizations.^[13]^ A preliminary assessment^[11]^ showed that mortality was statistically higher for patients presenting more than one complication (Fig. 1).

Thus, the primary endpoint was the in-hospital occurrence of a postoperative complication defined as the occurrence of at least 2 of the following postoperative events, derived from the Major Adverse Cardiac and Cerebrovascular Event composite criteria and the guidelines for mitral valve repair^[14]^:

1. use of inotropic and/or vasoactive agents for more than 24 hours,
2. new onset of atrial fibrillation requiring treatment and/or prolonged use of cardiac pacing,
3. acute renal failure, according to the R class of the risk, injury, failure, loss of kidney function, end-stage kidney disease classification,
4. mechanical ventilation >24 hours, and
5. postoperative sepsis defined as a clinical suspicion of infection confirmed by microbiological documentation or favorable evolution of biological and clinical parameters through empirical antibiotic therapy.

Secondary endpoints included length of hospital stay and in-hospital mortality.

### 2.5 Statistical analysis

Preliminary assessment^[11]^ showed that 50% of patients over 80 years old versus 30% of the younger patients presented with more than one postoperative complication. Using a ratio of sample sizes in group 1/group 2 of 4:1, a sample of 315 patients would be required to detect a difference (type I error: 0.05, and type II error: 0.20). Distribution of variables were tested by the Kolmogorov–Smirnov test. The results are expressed as mean ± standard deviation or median [extremes] according to their distribution. The occurrence of postoperative complications were compared between groups by Fisher exact test for qualitative variables and Student *t* test or Mann–Whitney *U* test for quantitative variables, depending on their distribution. Pre- and peroperative variables were selected for the logistic regression if *P* < 0.10.

Logistic regression was performed with postoperative complications as the dependent variable and body mass index, smoking, hypertension, diabetes, hypercholesterolemia, peripheral arteriopathy, chronic pulmonary disease, chronic kidney disease, history of myocardial infarction, New York Heart Association class ≥2, left ventricular ejection fraction <45%, pulmonary hypertension, associated ischemic heart disease, associated surgery, and surgery duration as the independent variables. Model performance was evaluated by the Nagelkerke adjusted *R*^2^ statistic, the c-index (discrimination), and the Hosmer Lemeshow goodness-of-fit test (calibration). The logit predicting probability of occurrence of postoperative complications (propensity score) was used to match patients according to age (under 80 years old and 80 years old and over; 2:1 matching). Then, univariate and multivariate analyses were performed on the matched population with occurrence of postoperative complication as the dependent variable.

All the tests were bilateral and *P* < 0.05 was considered to be significant. Statistical analyses were carried out using R Statistical Software (Foundation for Statistical Computing, Vienna, Austria).

## 3 Results

**Figure 2:**
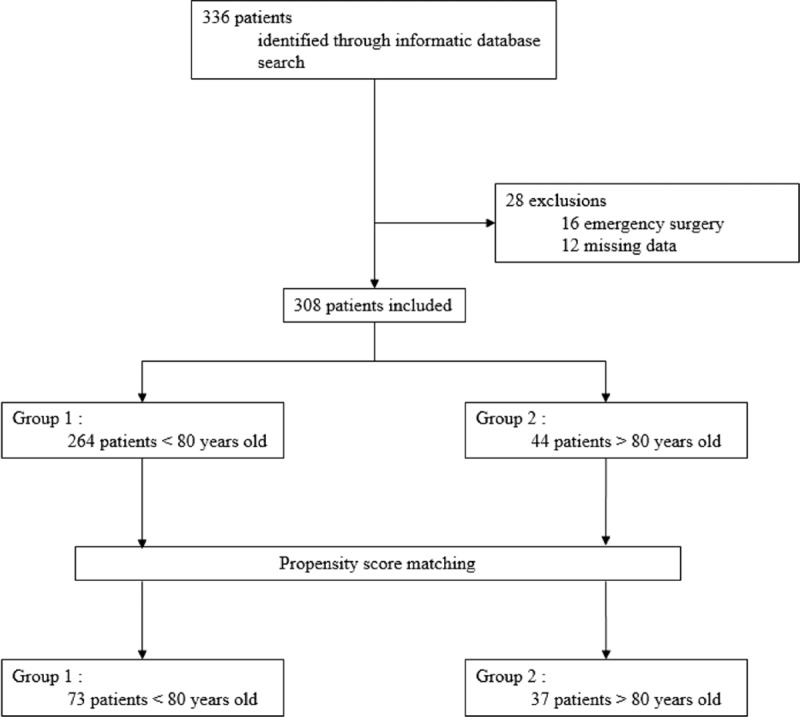
Study flow chart.

During the study period, data from 308 patients who underwent isolated or combined mitral valve repair were analyzed (Fig. 2).

**Figure 3:**
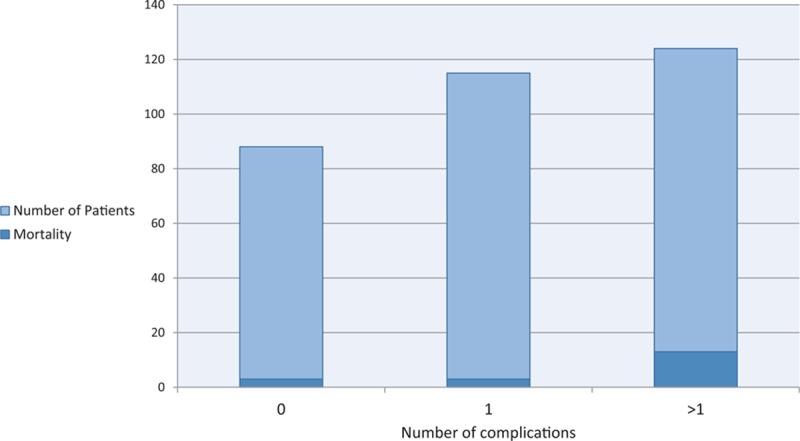
Incidence of complications in the total cohort.

The main characteristics of the patients are reported in Table 1. Cardiovascular risk factors were found in 287 patients (93.2%; 95% confidence interval [90.4–96.0]). Figure 3 shows the distribution of postoperative complications among the 308 patients studied. Postoperative mortality ranged from 3.5% without postoperative complications to 2.7% (*P* = 0.297) and 11.7% (*P* = 0.005) with one and more than one complication, respectively.

**Table 1:**
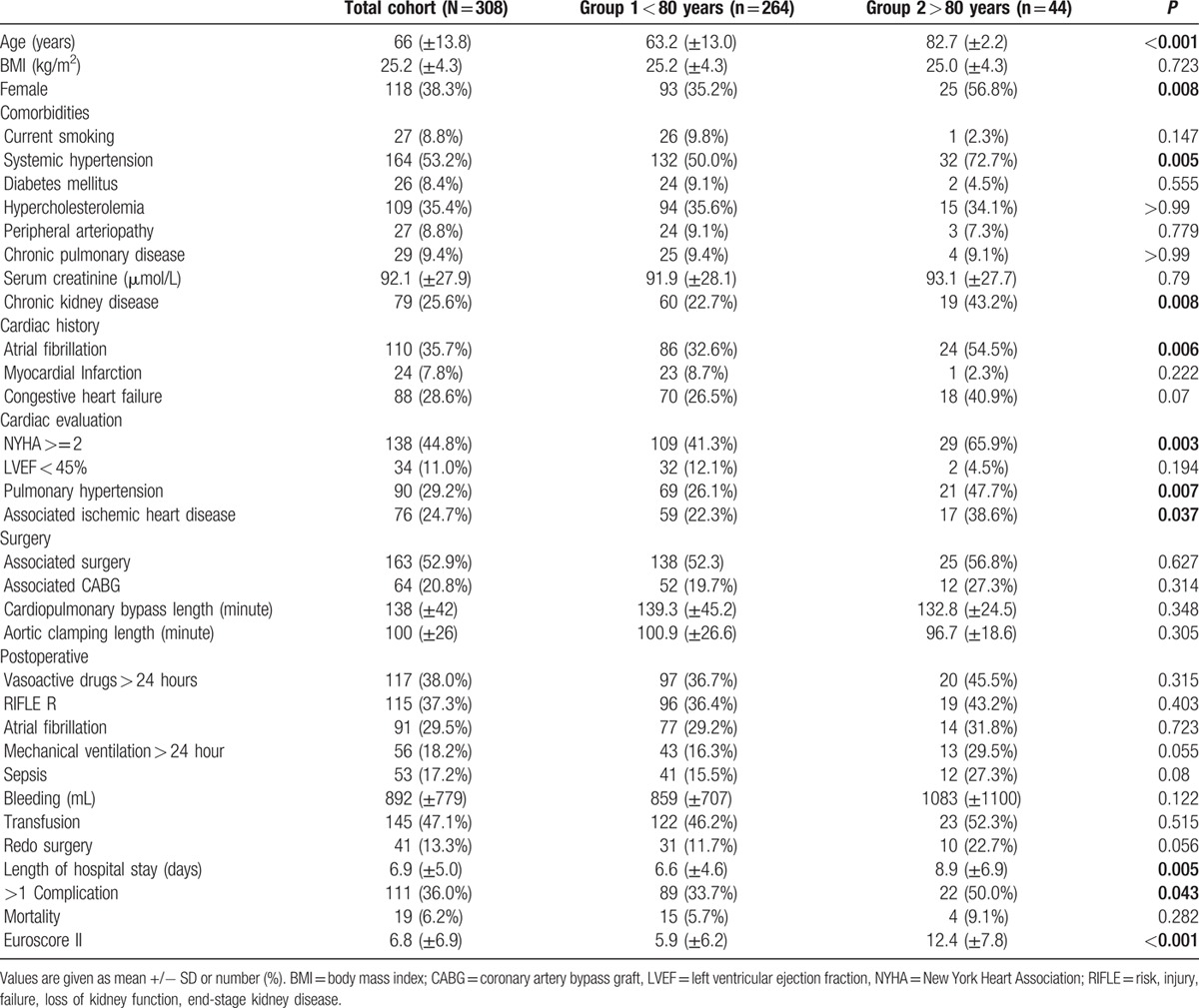
Patient characteristics according to age.

### 3.1 Age classification results

Comparing patients under and over 80 years old (Table 1), age-related conditions such as systemic hypertension, chronic kidney disease, and atrial fibrillation were found more frequently among older patients. Preoperative cardiac evaluation showed that older patients more frequently had pulmonary arterial hypertension (*P* = 0.007), associated ischemic heart disease (*P* = 0.037), and more patients presented with a New York Heart Association classification ≥2 (*P* = 0.003). Patients over 80 years old had more postoperative complications than the younger patients (50.0% vs 33.7%; *P* = 0.043). Their hospital stay was longer (8.9 ± 6.9 vs 6.6 ± 4.6 days; *P* = 0.005), but their reported mortality was not significantly different with G1 (9.1% vs 5.7%; *P* = 0.282), despite a predictive higher Euroscore II (12.4 ± 7.8 vs 5.9 ± 6.2; *P* < 0.001).

### 3.2 Complication propensity score results

The propensity score was built after comparison between patients who presented with postoperative complications and those who did not (Table 2). Patients with postoperative complications had more preoperative morbidities, cardiac history, and a poorer preoperative cardiac evaluation. They more frequently had associated surgeries and their surgeries were longer. The observed mortality was higher in patients with more than one postoperative complication (11.7% vs 3.0% *P* = 0.005).

**Table 2:**
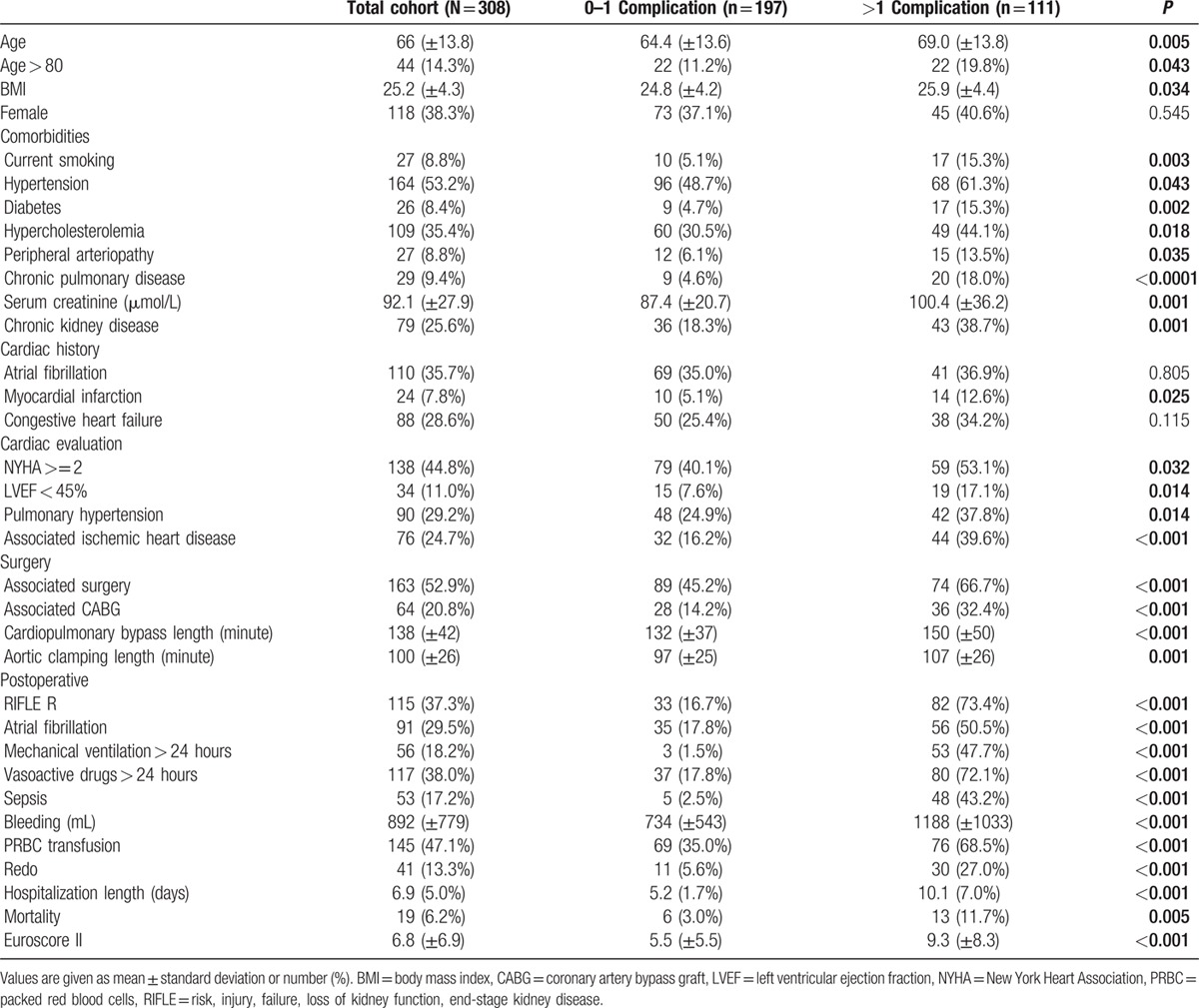
Comparison of patients with and without more than one complication.

When including univariate predictors in a multivariate analysis, 6 of them emerged as independent risks factors of complications (Table 3).

**Table 3:**
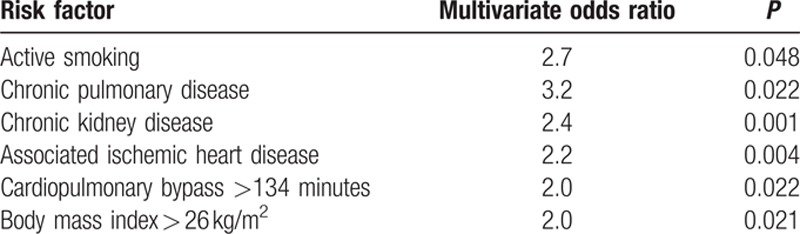
Odds ratio of multivariate risk factors of postoperative complications.

**Figure 4:**
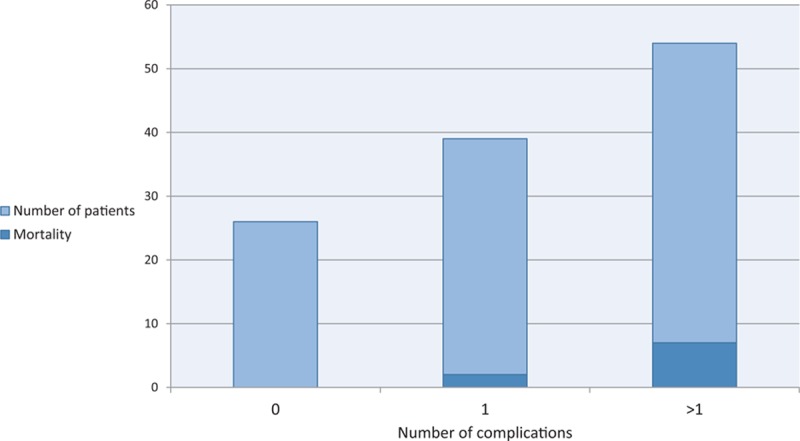
Incidence of complications in the matched population.

**Figure 5:**
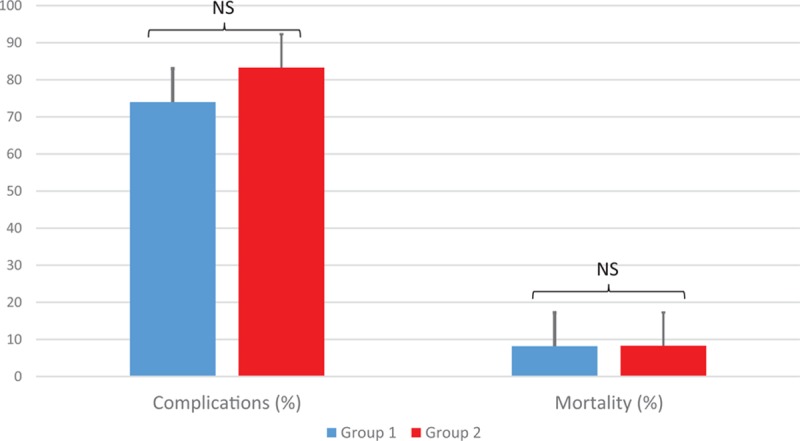
Postoperative outcome before (group 1) and after 80 years old (group 2).

The matching procedure resulted in a new population, described in Table 4 and Fig. 4. No difference regarding comorbidities was found after matching. When comparing patients under and over 80 years old, no significant difference was found, neither in terms of morbidity nor mortality (Fig. 5).

**Table 4:**
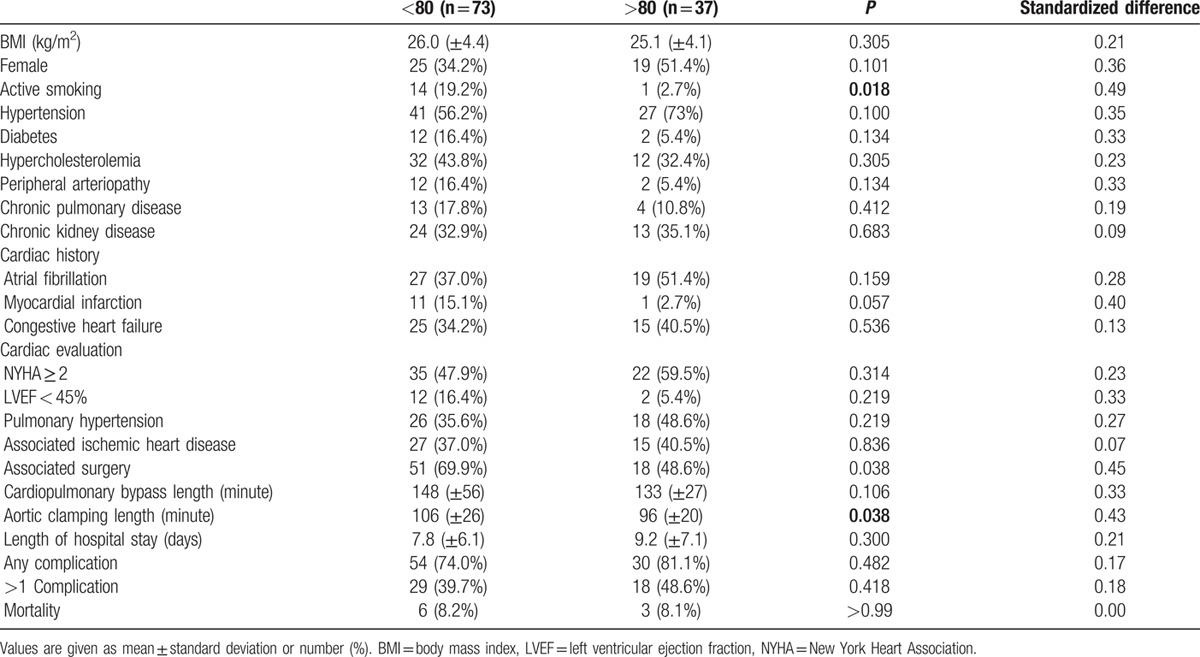
Comparison of old and young patients after propensity score matching.

## 4 Discussion

In the descriptive analysis, older patients presented with more postoperative complications. However, age is not an independent risk factor of morbidity and mortality in our study. Using the propensity score, no significant difference was found, neither in terms of morbidity nor mortality between younger and older patients. Thus, the high-risk morbidity may depend more on the comorbidities than the patients’ age.

Many precautions were taken to avoid bias. The single-centered design of this study ensured the uniformity of medical and surgical care for the whole cohort. The use of a propensity score to reduce selection bias is now widely recommended and provides a robust comparison.^[15]^ Finally, the primary endpoint was defined as the occurrence of at least 2 postoperative events due to the extremely high incidence of atrial fibrillation and acute kidney injury after cardiac surgery.^[16]^

Preoperative risk assessment is mandatory to provide patients with full information and to adapt the surgical strategy. It includes widely used scores (Society of thoracic surgeons,^[17]^ Euroscore II^[18]^) taking age into consideration. However, those scores poorly predict postoperative outcomes for medium-risk patients.^[8]^ One plausible explanation is the small number of old patients in the cohorts used to build and calibrate the scores.^[18]^ The effect of comorbidities such as atrial fibrillation and chronic kidney disease on patient postoperative outcome have been demonstrated in several studies, particularly in cardiac surgery.^[19–22]^ Older patients have more comorbidities, thus age might not be an independent risk factor, and predictive scores should not include it. The respective weights of these comorbidities need to be reevaluated.

Although comorbidities cannot be modified, even with optimized medical treatment, we identified 3 independent risk factors that could be modified: active smoking, associated ischemic heart disease, and cardiopulmonary bypass length. First, active smoking should be stopped before cardiac surgery. History of smoking was not among the univariate predictors of complications in our study and other studies showed that 6 to 8 weeks’ abstinence improved the outcome.^[23]^ Second, associated ischemic heart disease was found in 40% of the patients who presented with postoperative complications. Preoperative percutaneous angioplasty would have the double benefit of improving myocardial perfusion before cardiopulmonary bypass and of reducing surgery time. However, percutaneous techniques are not always technically feasible, especially for coronary bifurcation lesions and extended stenosis,^[24]^ and raise the problem of surgery under antiplatelet therapy. Third, cardiopulmonary bypass duration could be shortened in various ways. In our study, the proportion of combined surgeries was high in both groups. Reducing combined surgery among high-risk patients might improve the outcome and reduce the surgical duration, as suggested by Craver et al.^[25]^ This reduction could be achieved through the development of mini-invasive treatment,^[26]^ percutaneous treatments,^[27]^ or hybrid-procedures, which provide additional benefits. The absence of sternotomy with mini-invasive techniques might impact postoperative respiratory comfort and pain, reducing the consumption of morphine derivatives and length of hospital stay.^[28]^ Percutaneous trans-femoral valve replacement does not even require general anesthesia, further reducing the postoperative risks.^[27]^

Our study had several limits. First, it was a single-center retrospective study, which required an extended inclusion period, during the patient treatment evolved, both surgically and from the point of view of perioperative anesthesia. However, the change affected both groups. Second, we studied all types of mitral valve repair, with no distinction of indication (effects of an aortic or myocardial pathology, effect on the right cavities requiring an associated procedure). Third, Fried et al^[29]^ developed a concept of frailty phenotype among elderly patients, which leads to increased mortality and morbidity after cardiac surgery,^[30]^ but that was not assessed in this study, nor were preoperative nutritional status or postoperative cognitive dysfunction. Those are a main concern among elderly people as they may condition their postoperative recovery and impact their quality of life. Therefore, an assessment of geriatric frailty should be incorporated in preoperative evaluation by surgeons, cardiologists, and anesthesiologists.

Finally, no medium or long-term data were collected which would certainly have affected the mortality observed. Relative to this study data, there was no difference between old and young patients regarding hospital mortality. However, the occurrence of isolated postoperative renal failure is enough to increase mortality in the medium and long-term.^[31]^ Observation of a major increase in morbidity should involve increased long-term mortality, consistent with previous studies.^[32,33]^

## 5 Conclusions

Mitral valve repair is subject to frequent postoperative complications and mortality, especially in elderly subjects. This is probably due to the more frequent comorbidities observed in this population. Preoperative risk assessment has to be modified and surgical strategies should evolve with the patient's preoperative comorbidities rather than age itself.
